# Commentary on ‘Irisin Attenuates Ventilator‐Induced Diaphragmatic Dysfunction by Inhibiting Endoplasmic Reticulum Stress Through Activation of AMPK’

**DOI:** 10.1111/jcmm.71221

**Published:** 2026-06-01

**Authors:** Wei Li, Hui Yang, Jia Li, Xueqin Xu

**Affiliations:** ^1^ Department of Critical Care Medicine Shiyan Renmin Hospital, Hubei University of Medicine Shiyan Hubei China; ^2^ Department of Anesthesiology Xiang Yang Hospital of Traditional Chinese Medicine Xiang Yang Hubei China; ^3^ Institute of Anesthesiology & Pain (IAP), Hubei Provincial Clinical Research Center of Central Nervous System Repair and Functional Reconstruction, Taihe Hospital, Hubei University of Medicine Shiyan Hubei China

Dear Editor,

Recently, we read with interest the article entitled ‘Irisin attenuates ventilator‐induced diaphragmatic dysfunction by inhibiting endoplasmic reticulum stress through activation of AMPK’ by Zhang et al., published in the *Journal of Cellular and Molecular Medicine*, in 2024. This study convincingly establishes that prolonged mechanical ventilation (MV) precipitates ventilator‐induced diaphragmatic dysfunction (VIDD), a condition whose severity escalates in direct proportion to the duration of support. Mechanistic analyses indicate that prolonged MV triggers a cascade of pathological events characterised by suppressed circulating irisin concentrations, intensified endoplasmic reticulum stress, elevated oxidative burden, and increased cellular apoptosis. Collectively, these alterations drive diaphragm atrophy and significantly impair contractile performance. Exogenous irisin supplementation can inhibit endoplasmic reticulum (ER) stress response by activating the AMPK pathway and alleviate VIDD, thus playing a protective role. This discovery provides an important theoretical basis for the application of irisin in critically ill patients, especially in ventilator‐dependent patients.

Upon reading, we found that the study had methodological shortcomings in verifying mechanisms, mainly due to the absence of AMPK loss‐of‐function rescue experiments. According to the study, irisin intervention reversed the decrease in phosphorylated AMPK (p‐AMPK) levels caused by MV, suggesting that irisin protects against VIDD through the activation of AMPK. Despite the obvious correlation between irisin intervention and increased AMPK phosphorylation, the authors did not implement AMPK inhibition or knockdown groups to demonstrate that irisin affects ER stress through the AMPK pathway. In the absence of rescue experiments, the possibility remains that irisin inhibits ER stress through other signalling pathways and consequently ameliorates VIDD, while the observed elevation in p‐AMPK levels may represent a secondary compensatory mechanism. Therefore, the association between AMPK activation and ER stress cannot be definitively established as causal.

To confirm the Irisin‐AMPK‐ER stress regulatory axis, we suggest incorporating an AMPK blockade strategy into the experiment. Specifically, experimental groups receiving MV plus irisin combined with either an AMPK inhibitor (Compound C) or AMPK gene silencing should be included. To rigorously evaluate our postulate, we conducted a comparative analysis of critical metrics across four experimental cohorts: the control group, the MV model group, the MV + irisin group, and the MV + irisin + AMPK inhibition group. The assessed parameters encompassed diaphragmatic contractile function, fatigue resistance, myofiber cross‐sectional dimensions, levels of atrophy‐associated proteins (MuRF‐1, Atrogin‐1), oxidative stress markers (ROS, MDA, GSH‐Px), apoptotic regulators (Bax/Bcl‐2 ratio), and endoplasmic reticulum (ER) stress indicators (GRP78, GRP94, CHOP). We posited that blocking AMPK signalling would substantially diminish the cytoprotective benefits conferred by irisin, thereby abolishing its capacity to suppress ER stress. Such rescue experiments are necessary to confirm that irisin inhibits ER stress and mitigates VIDD in an AMPK‐dependent manner.

In conclusion, the current research is significant and valuable. However, we also suggest strengthening the mechanism verification to further clarify that irisin plays a protective role by regulating the AMPK pathway and mediating the inhibition of ER stress. We hope that this perspective will be useful for future research.

## Author Contributions

**Wei Li:** writing – original draft, writing – review and editing. **Hui Yang:** writing – review and editing, methodology. **Jia Li:** conceptualization, methodology. **Xueqin Xu:** conceptualization, writing – review and editing.

## Funding

This work was supported by Shiyan Municipal Bureau of Science and Technology, No. 24Y094, No. 25Y083.

## Ethics Statement

The authors have nothing to report.

## Conflicts of Interest

The authors declare no conflicts of interest.

## Linked Articles

This article is linked to Zhang et al. papers. To view this article https://doi.org/10.1111/jcmm.18259.

## Data Availability

No datasets were generated or analysed during the current study.

